# “This is how I'm going to do it, but this is not how you're going to do it”: the expectation gap between student paramedics and mentors in East and Central Scotland

**DOI:** 10.1186/s12909-024-05319-z

**Published:** 2024-04-03

**Authors:** Megan E. Worsfold, Clare Jouanny, Ela Hamer, Stian A. Mohrsen, Patryk Jadzinski, Mick Harper

**Affiliations:** 1 University of Stirling, Stirling, Scotland; 2 Hampshire Hospitals NHS Foundation Trust, Winchester, UK; 3 University of Portsmouth, Portsmouth, England

**Keywords:** Educator, Mentoring, Paramedic, Placement, Student, Expectations

## Abstract

**Background:**

The role of paramedics has expanded significantly over the past two decades, requiring advanced skills and education to meet the demands of diverse healthcare settings. In 2021, the academic requirements for paramedics were raised to a bachelor’s degree to align with other registered professions. The limited evidence on effective paramedic practice education necessitates a novel or new examination of unique learning methods, emphasising the need to establish effective learning relationships between mentors and learners to enhance professional respect and support achieving learning outcomes. This study aimed to investigate expectations between student paramedics and their mentors, focusing on the learning dynamics within paramedic education.

**Methods:**

This qualitative study used purposive sampling to recruit participants from two distinct cohorts: student paramedics from the University of Stirling and Practice Educator Mentors from the Scottish Ambulance Service. Focus groups were conducted to illuminate comprehensive insights into participants' expectations regarding practice education and their respective roles in the learning process. Codebook thematic analysis was used to assess the alignment of these expectations.

**Results:**

Findings illustrate important challenges within practice placement across learning paradigms and highlight the attitudes surrounding the integration of higher education and expectations of practice placements. These challenges encompass systemic barriers, including the support provided to mentors as they assume increased responsibilities and barriers that deter qualified staff from initially undertaking this role.

**Conclusion:**

The study aimed to assess expectations between practice educators and students within the paramedic profession in Scotland. The methodology effectively identified key themes from comprehensive data, marking the first primary research in this field. There are disparities in learning styles, expectation measurement, and attitudes toward higher education during practice placements, which could significantly impact the teaching and assessment processes. The findings suggest increased support for practice educators, educational programs addressing challenges of mentorship, and stronger links between higher education institutes and the Scottish Ambulance Service. Further research is needed to understand the extent of the expectation gap, how expectations evolve, and to develop strategies to address disparities.

## Background

In the last 20 years, the paramedic profession experienced/observed an exponential growth with the paramedics’ scope of practice developing beyond the emergency ambulance service into wider healthcare settings such as primary care, injury treatment centres, forensic services, and hospital care [1–3]. Simultaneously, specialist and advanced paramedic roles, both clinical and non-clinical, are increasing in workforce planning, requiring postgraduate skills to enable practitioners to engage with research and advanced leadership [4, 5]. In order to meet the requirements of diversifying professional demands, the 2013 Paramedic Evidence-based Education Project (PEEP) report emphasised the role of broader theoretical and systems-based learning, in contrast to the historical practice-focused nature of ambulance work [6]. In 2021 the regulator of paramedics and other allied health professions (AHPs) across the UK, The Health and Care Professions Council (HCPC), raised pre-registration qualification requirements to a minimum of a Bachelor’s degree (BSc), to meet minimum academic requirements and align the standard of paramedic education with other registered professions [7].

More time allocated to theory and increasing numbers of student paramedics competing for ambulance service placements have highlighted the importance of effective practice-based learning to bridge theory and practice [8]. Supervised practice education has been extensively studied and refined in medicine and nursing with proven methods and frameworks extrapolated, largely successfully, across other healthcare professions [9, 10]. However, the current evidence regarding paramedic practice education is limited or arguably, outdated [11, 12]. While some concepts can be inferred from successful practices in related professions, the authors understand the field of paramedicine to carry a set of unique features that juxtapose effective learning including those listed in Table 1.

**Table 1:** Authors experiences of unique features to effective learning

**The acuity of dispatch and response**	Student paramedics often respond to situations where high acuity is either manifested by the clinical condition or perceived by patients or bystanders, affecting stress and concentration
**Dynamic and hostile environments**	The environments paramedics frequently operate in are dynamic and occasionally hostile including unknown residents, public areas, crowd events, and moving vehicles
**Exposure to trauma/inaccessibility of rest and facilities**	Students and their mentors may find it challenging to avoid exposure to traumatic situations and have limited opportunities for rest, affecting their ability to engage in focused learning
**Working in small teams with limited resources**	Paramedics often work in small teams with constrained resources, which can impact the availability of educational opportunities
**Limited time/access to learning space**	There may be limited time and suitable spaces for essential activities such as debriefing, meaningful learning conversations, and research

**Fig. 1:**
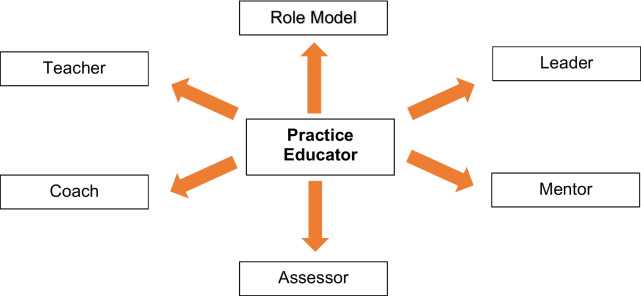
Roles of a practice educator - image adapted from Clarke, 2020 [13]

Endorsed by The College of Paramedics, who emphasise that the term 'mentor' has been widely adopted from other healthcare professions, referring to a person who teaches or supervises a mentee. However, as the profession has expanded in a unique direction, so has the role within paramedic practice education, leading to its recognition as multifaceted and more recently designated title as 'practice educator’ (see Fig. 1) [13].

Furthermore, a large proportion of the existing evidence exploring practice learning focuses on personal and professional attributes of supervisors, or student-perceived barriers to learning used to define “good mentorship” where the responsibility for learning is shifted onto the mentor [14, 15]. Others debate that the learning experience is a relationship in which the learner and mentor share the responsibility for effective learning by contributing to, and reflecting on, mutual discussion and feedback [10, 16].

Considering these challenges and the limited time allocated to practical education, learners and their mentors must be able to establish a professional rapport, and studies conclude that failure to establish effective learning relationships contributes to negative impacts on student paramedics’ learning [17, 18]. Alignment of expectations between supervisor and learner has proven to enhance respect, professionalism, and mutual trust. These are all crucial to effective learning, but failure to align can result in a dysfunctional relationship negatively affecting the engagement of both supervisor and learner [17].

The paucity of literature describing and exploring expectations and attitudes between paramedic practice educators and learners makes it challenging to interpret behaviours and experiences within the practice placement setting and plan for effective learning. To address this knowledge gap and contribute to the future preparation of practice-based learning for both student paramedics and their Practice Educator Mentors (PEMs), we undertook a small-scale qualitative study. The primary aim being to explore the expectations of mentorship between student paramedics and their PEMs across the regions of Forth Valley, Fife, and Tayside in Scotland.

## Methods

### Recruitment

A purposive sampling strategy was used to recruit a sample of students and PEMs who met predefined inclusion and exclusion criteria (see Table 2). In the time the study was advertised, and data was being collected, eight participants agreed to take part in the study. Participants volunteered in response to information posters and emails distributed in workplaces and educational institutions, they then received information sheets and consent forms before deciding to participate. Randomisation within-group sampling was used to limit sample size where appropriate to give each recruited participant an equal chance of inclusion.

**Table 2:** Inclusion and exclusion criteria

Selection criteria		
**Student paramedics**	Inclusion	Enrolled on UoS BSc Paramedic Science
		Undertaken ≥ 1 practice placement
	Exclusion	Leave of absence during study period
**Practice educators**	Inclusion	Registered paramedic
		Qualified ≥ 1 year
		Based in UoS catchment area
		Any experience mentoring UoS BSc paramedic science student
	Exclusion	Previous experience mentoring BSc students elsewhere

Student paramedics were invited from the University of Stirling (UoS) BSc Paramedic Science programme. This was motivated by the fact that this was the academic base of the study team at the time, but more importantly that it was a newly established programme. This helped ensure that participants were not influenced or biased by the experiences of previous cohorts and allowed the capture of fresh, untainted perspectives. Students had to have completed a minimum of one practice placement module to provide a frame of reference and experiences on which to draw from during the focus groups.

Practice educators were HCPC registered paramedics invited from the Scottish Ambulance Service, specifically in the UoS catchment area for practical placements. As other areas of Scotland already established undergraduate programmes in preceding years, we sought to gain the experiences of novice educators.

### Data collection

From a recruited pool of ten volunteers, eight student paramedics were randomly selected using an online number generator. Two participants were in their first year of paramedic science and the remaining six were in their second year of paramedic science. All students were enrolled and actively studying on the Paramedic Science BSc programme at UoS. Using the same method of recruitment, a total of five PEMs were recruited initially, with three later withdrawing due to unknown circumstances. The remaining two PEMs had over five years of experience as qualified paramedics (Table 3).

**Table 3:** Summary of data collection participants’ clinical experience/placement experience

**Student Paramedics**	
Recruited	10
Included	8
Year 1 student	2
Year 2 student	6
**Practice Educator Mentors**	
Recruited	5
Included	2
Years of experience	> 5

Data was collected through semi-structured focus groups (see Table 4) conducted between June – July 2022. Focus groups address funding and time constraints, but also offer advantages such as facilitating participant engagement and provide a conducive environment for open discussion [19].The interview sessions, lasting up to one hour, were facilitated remotely through Microsoft Teams by research staff from the UoS who were independent of the Paramedic Science programme and the Scottish Ambulance Service. Facilitators were equipped with online briefings and debriefings, as well as a semi-structured questioning approach, enabling them to maintain focus while also providing flexibility for detailed exploration and follow-up questioning as needed [20]. Two student paramedic (SP) focus groups were conducted, each with four students and one facilitator, and one group was conducted with the two PEMs. Focus groups were audio recorded and transcribed verbatim by the lead researcher, allowing thorough familiarisation with the data pre-analysis. The lead researcher was also observing the focus groups passively to allow for documentation and subsequent analysis of non-verbal cues, which resulted in providing a comprehensive understanding of the data beyond the spoken words. To promote candid and open dialogue, focus groups were organised with up to four participants, and importantly, to minimise bias, student and paramedic educator groups were kept separate.

**Table 4:** Focus group schedule

Focus group schedules	
**Student focus groups**	What does the term ‘practice educator’ mean to you?
	What do you expect of your practice educator mentor?
	What do you think your practice educator expects of you?
	Whilst out on placement, how do you expect your practice educator to facilitate your learning?
	Do you feel your expectations are met?
	Do you feel your expectations have changed over time?
**Practice Educator focus groups**	What does the term ‘student’ mean to you?
	What do you expect of a student paramedic?
	What do you think your practice student expects of you?
	Whilst on placement, how do you expect students to facilitate their learning?
	Do you feel your expectations are met?
	Do you feel your expectations have changed over time?

### Data analysis

**Fig. 2:**
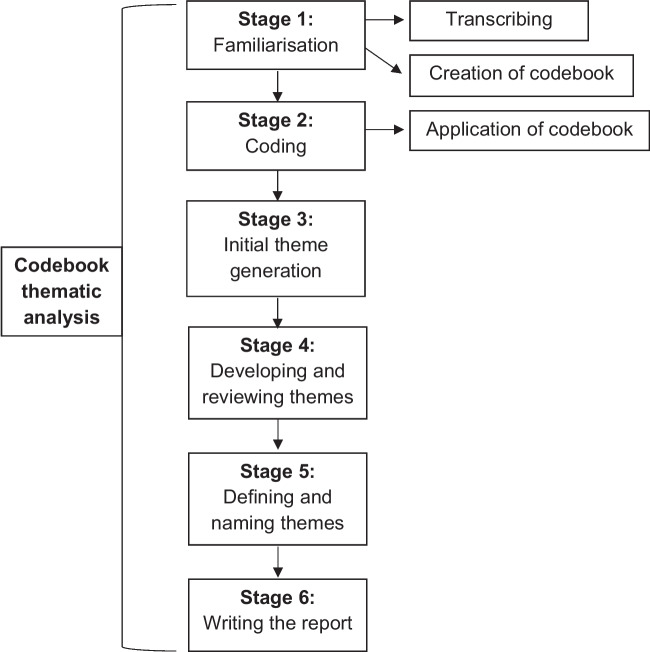
Steps of codebook thematic analysis [22]

Data was anonymised, transcribed, and analysed in NVivo by the lead researcher [17]. Themes that emerged were reviewed by a second researcher (SM) without requirement for any amendments. The application of codes from a codebook designed in the familiarisation stage of the data is a deductive approach, reducing time spent on coding whilst still accessing data through a qualitative lens [21]. The researcher being fully immersed in the data at this stage, and the transcription process is seen as a method of bracketing, to reduce the risk of preconceptions and predetermined confirmation that can be associated with codebook thematic analysis. Figure 2 provides a graphic representation of codebook thematic analysis, adapted from Braun and Clarke [17].

Analysis was driven by role theory which sets out that people hold positions within society that are paralleled with expectations of behaviours of self and others [18]. This allowed for exploration of expectations embedded within the role of the student and the practice educator and helped shed light on how these sometimes conflict or converge [19] Highlighting discrepancies in anticipated behaviours in the mentor-learner relationship led to improved understanding of sources for conflicting expectations. This method involves a recurring process of gathering data, analysing, and applying a codebook, before conducting further analysis until reaching a point of data saturation [22]. The data collection and analysis process involved acknowledging the role of the researcher. This reflexive approach enabled the lead researcher to critically reflect, applying introspection and self-awareness of bias throughout [23].

## Results

Fifteen codes were identified through initial coding before being refined and described into four major emerging themes (Table 5).

**Table 5:** Reviewing and defining themes

Codebook/Coding	Initial themes	Developing and reviewing	Naming and defining
- The role of a PEM - Attributes - Attitudes on University system - Understanding clinical ability - Communication - Mentoring experience - Differences in theory to practice - Relationships - What students believe is wanted from them - Suggestions of improvement - Practice assessment document - Meeting expectations - Experiences - Motivation to mentor - Evolvement of the service	- Applying theory to practice - Attributes - Teaching and learning styles - Relationship - Motivation to mentor - Mentor education - Attitude on university culture - Student clinical ability - Practice assessment document - Previous experience - Changes in expectation	- Attributes to effective learning - Academic paradigm - System barriers to effective learning - Changes in expectation - Previous experience	1. Attributes to effective learning 2. Academic paradigm 3. System barriers to effective learning 4. Changes in expectation

Throughout the focus group sessions, participants spontaneously offered suggestions for enhancing the teacher/learner experience, with a particular focus on improvements within the practice learning environment. These suggestions addressed possible solutions to existing challenges. It is important to note that this study did not specifically aim to explore recommendations for practice, and these suggestions did not align with the predefined themes for analysis. Consequently, they were excluded from the analysis conducted in this study. However, these suggestions may be of interest to policymakers or for future research purposes and are available from the main author on request.

### Theme 1: Attributes of effective learning

Among the themes identified in the data analysis process, the attributes of effective learning received the most extensive attention. Within this theme, various codes encompassing individual attributes, teaching and learning styles, relationships, and elements of mutual learning and communication were amalgamated. This comprehensive theme explored the various features that contribute to effective learning approaches and conducive learning environments. Attributes and communication were instrumental in providing insights into the expected level of engagement and the establishment of strong connections within working relationships, which were widely recognised as pivotal for both effective learning and teaching styles. Notably, student participants perceived their relationships with PEMs as professional, yet they also viewed them as friendly figures. Conversely, PEMs tended to see their role in a more parental light, fostering a different kind of connection with their students.

*“It is like working with kids, you know…” [PEM 1]*

Also referring to the act of taking on a student when another PEM is on annual leave as ‘*babysitting’* [PEM 2], perhaps denoting their role to be overseeing or preventing mistakes and failure as opposed to teaching and nurturing good practice.

PEMs expressed a clear expectation that student paramedics should be eager to immerse themselves in the learning process and actively expand their own knowledge. One PEM emphasised this by stating:

*"I want someone who is going to ask the questions. I want someone who is going to be keen to learn" – [PEM 1]*

Students also recognised the importance of keenness as an attribute for effective learning. However, their perspective appeared to involve a slightly more passive role in the learning process. As articulated by one participant:

*"I think just somebody who's willing to think about you and your learning and to invest in that, who will take the time ... who's willing to then come back and say, 'Let's talk about this?'" - [SP 4]*

Both groups valued enthusiasm for learning, but it is striking that students expected their mentors to take an active role in engaging with them, investing in their educational development, and facilitating discussions. This reflects a desire among students for guidance and mentorship throughout their educational journey.

Students’ experiences varied between the time and place where they undertook practice learning. They identified that applying an appropriate level of pressure and encouraging knowledge exploration were effective strategies for extending their learning comfort zones. However, the success of these strategies appeared to be contingent on the presence of mutual respect between students and their PEMs. One student discussed an experience where they were made to feel unwelcome when their PEM left them unattended for an extended period:

*“...they got a call, and I was walking out the door and had all my kit on... and they drove off and left me at the station for two and a half hours and to me, that was a pretty clear sign. It made me feel very unwelcome.” – [SP 6]*

This behaviour had implications surrounding the relationship between the student and their PEM. However, two other students praised their PEMs for the enthusiasm and opportunities they were provided, including the great relationships they built up. The framing of relationships through the sharing of knowledge, experiences, and mutual values frequently came up in both data sets.

### Theme 2: Academic paradigm

The development of a BSc Paramedic Science programme in Scotland has provoked ambivalent feelings amongst both cohorts with scepticism found among qualified paramedics:

*“I think there are people that just do not want to put in the effort...a lot of staff have been in the job for several years and argue that well, it's not an academic job” - [PEM 2]*

This theme, was also reflected amongst students in their experiences of practice placement:

*“Sometimes it's like they don't like the new system, they don't trust the system...” – [Student 3]*

The same student compared the previous changes in other health care sectors to the more recent changes in paramedic practice, and shared a positive opinion surrounding these changes:

*“This happened with midwifery and nursing they both changed, and I feel like for the better to be honest” – [Student 3]*

Expectations surrounding the application of theory to practice surfaced opinions of how ‘academic’ the role of a paramedic is perceived to be. Students emphatically believed that the role of a paramedic has evolved, and they expected qualified staff to be accessible for both theoretical and practical inquiries:

*“Someone that you can almost go to for advice if you need and be it placement or be it the education kind of side about the theory side of it, just someone you could go to and bring up concerns bring up problems” – [SP 3]*

However, students observed that this ideal was not always realised in practice. They recounted instances where the integration of theory into practice was not only absent but also met with resistance. In these experiences, they encountered defensive behaviour from some qualified staff members who appeared reluctant to incorporate new and up-to-date theoretical knowledge:

*"There's a common phrase: 'This is how I'm going to do it, but this is not how you're going to do it. This is not the way you're taught...'" – [SP 4]*

These discussions focus on attitudes within the contemporary culture of paramedicine. Students articulated their expectations regarding engagement with the academic program from both students and educators, emphasising how this engagement could influence the learning and teaching that occurs in a practice-based education environment. Furthermore, these conversations highlighted a lack of understanding about the mutual benefits that can result from effectively integrating theoretical knowledge into paramedic practice.

### Theme 3: System barriers to effective learning

This study unveiled several barriers to effective learning, encompassing issues related to the education of PEMs and associated communication challenges. One prevalent barrier was the marked lack of communication and engagement concerning academic system knowledge, particularly in the context of assessing students and PEMs using the practice assessment document (PAD). This lack of clarity led to disparities in expectations of students' clinical abilities, resulting in unequal learning opportunities, as described by one student:

*"There's a lack of consistency... because you have mentors who allow some students to do almost everything, while others won't even let you measure blood pressure, you know?" – [SP 1]*

Furthermore, the study highlighted the stress experienced by qualified staff, compounded by the pressures of additional paperwork and responsibilities. These staff members felt inadequately supported and insufficiently educated by the system that surrounded them:

*"Why should I bother, then, if the service isn't bothered? Why should I be?" – [PEM 1]*

Students were aware of this stress within the ambulance service, and they reported experiencing feelings of guilt as a result. They believed that this stressful situation could potentially have been avoided:

*"Who decided that being a paramedic would involve a university route now... they throw you into ambulance service placements, and the ambulance service is like, ‘What?’ Like, ‘we were not prepared for this’, and then no one really takes responsibility for it. It's as if they've introduced this new way of learning without the necessary support and infrastructure" – [SP 8]*

Collectively, these barriers hindered the establishment of a positive learning and teaching environment. Students reported that the lack of engagement within the service had repercussions on their motivation:

*"They need to be motivated because, at the end of the day, if they're not motivated, it can affect our motivation too" – [SP 1]*

The paramedic role, already characterised by its unpredictability, poses numerous challenges to practice placements. However, the reporting of these additional barriers could further complicate the task of creating an effective learning environment.

### Theme 4: Changes in expectation

Both students and PEMs were specifically asked to reflect on whether their expectations had evolved over time, be that due to an increasing experience in practice, a developed understanding of the university system, or growing knowledge. This theme also sought to capture any notable expectations from second-year students that might be comparable to those of first-year students. Although no third-year students were recruited, second-year students discussed how they anticipated their expectations would rise and be accompanied by various challenges. They also discussed their perceived expectations from mentors during this phase.

*“I also think us going into the third year out on placement. I do think that they will expect – they will have really high expectations. They will expect us to be able to run a job.” – [SP 4]*

Second-year students disclosed that they had significantly adjusted their expectations since the beginning of their course, viewing this as a learned mechanism to enhance their learning experiences and environments:

*“Now I go in completely blank ... that's kind of one of the only ways as a student you can kind of get over the negativity and just go forward because placement is what you put into it and like obviously what people on the opposite side put in like makes a big difference... I have lowered my expectations considerably since the first day” - [SP 8]*

This theme also delved into the factors influencing these changes in expectations. Students appeared to gauge their expectations based on their evolving clinical abilities:

*“...as we develop our kind of skills as well, like my expectations of my next placement are going to be different from expectations of my last placement. And because I know more, and I can do more... it's a very evolving thing” – [SP 2]*

In contrast, mentors articulated that they had to tailor their expectations based on their prior experiences, whether due to a lack of understanding of clinical abilities or otherwise:

*"I think it all depends on their background... I think when you get the students who are younger or haven't really had that kind of life experience, your expectations change" – [PEM 2]*

This implies that expectations not only evolve over time but also vary according to the individual's confidence, experience, and comprehension of the field.

## Discussion

### Attitude towards higher education

Analysis shows that students hold an expectation of PEMs as experienced individuals who should exude confidence in their knowledge while maintaining a commitment to staying current with their practice. However, they articulate that they often encounter defensive and resistant behaviour among PEMs, who appear reluctant to adapt and assume additional responsibilities related to teaching. These behavioural patterns mirror findings from prior research, which coupled with conclusions from the Paramedic Evidence-based Education Project (PEEP), prompted the HCPC to elevate registration requirements to standardise paramedic education programs [6, 20].

A theme that emerged prominently from the students' narratives is the distinction between PEMs who have undergone university-based paramedic education and those who have received vocational, practice-based training within the service. Students consistently noted the value of PEMs with a university education background, emphasising that they bring a unique perspective and contribute to a more positive and proactive learning environment. This aligns with existing evidence, which suggests that qualified staff who have completed their paramedic education at a university often draw upon their own experiences with mentors—both positive and negative—to create a supportive and constructive learning atmosphere for current students during practice placements [22–24].

Despite these positive changes, resistance to change is apparent in the evidence among PEMs in Scotland, seemingly prioritising their own prior experiences over the new knowledge imparted in university settings. Effective practice education relies on continuous professional development on behalf of the PEM to strengthen both clinical and operational practice, but also the ability to facilitate learning [10]. Both students and PEMs describe power dynamics complicating learner-mentor relationships in the practice learning environment. This phenomenon could be attributed to systemic barriers but may result from a lack of awareness regarding the higher education pathway to paramedic registration, coupled with a failure to understand the significance of nurturing the next generation of paramedics and the mutual benefits it offers.

### System barriers

The lack of a supporting infrastructure reported as creating barriers for qualified staff, resulting in a lack of motivation to step into a PEM role. Staff members report an onerous set of responsibilities and requirements associated with the PEM role that are inadequately reflected in terms of scheduling and financial compensation. Consequently, those who have taken on this role often feel unsupported and report feeling ‘stressed’ resulting in a detrimental impact on both their motivation and job satisfaction, sentiments echoing previous literature [25]. Analysis shows that this situation has not only prompted qualified staff to step away from the PEM role but is also reported as engendering a negative atmosphere and attitude that permeate the learning environment.

In some instances, PEMs report having pre-emptively conveyed their dissatisfaction to discourage students from pursuing this career path and students, whilst students articulate that they have keenly observed the constraints faced by their mentors which has left them with a sense of guilt and burden. The impact of guilt is known to reduce self-esteem, create barriers to goal achievement, and even manifest as physical symptoms such as anxiety [26]. This underscores the dual impact of the lack of infrastructure—staff members experience negative stress, while students grapple with the emotional toll of guilt and burden.

### Learning styles

Students and PEMs express that enthusiasm and investment as two crucial attributes for a positive learning environment. These attributes are typically assumed to be inherent in individuals volunteering to become PEMs and in students enrolling in university courses. Specifically, student paramedics expressed a desire for investment from their PEMs through effective communication and a willingness to challenge them by probing their knowledge and identifying areas for improvement. As such, students seemed to prefer a more passive role, where they were not solely responsible for asking questions, possibly to alleviate the perceived burden on their mentors. However, it's important to note that this passive learning style may not be the most effective approach for adult learners [27]. Conversely, PEMs described a teaching style in which learners take the initiative to ask questions and demonstrate their independence in the learning process. This approach places students in a more active role while positioning PEMs as more of a guide or facilitator, in keeping with contemporary andragogy [28, 29].

It is apparent from the data, that not only are both roles awaiting the other to communicate, but both roles are trying to reduce their own workload, and by doing so, creating a relationship based on incorrect learning styles. For the practice learning environment to be effective, it is important both student and PEM are aware of their own and each other’s learning styles [30, 31]. This instils a need for the flexibility to adapt practice to suit individual learning styles in building a learning relationship that will positively affect students, PEMs, and the practice area [18].

### Assessment in practice

The lack of understanding regarding the Practice Assessment Document (PAD) potentially signifies a communication gap between higher education institutions and the ambulance service. However, it can also be interpreted as a lack of engagement on the part of PEMs with the available guidance. PEMs appear uncertain about the clinical abilities of students at different stages of their education, leading to unequal opportunities and, at times, difficulties in connecting theoretical knowledge to practical application.

Students expected there to be some level of education or training provided to PEMs before they take on the role. In contrast, PEMs acknowledged their unfamiliarity with academic processes, including the PAD, even though they were more familiar with the mentoring environment through internal trainees. It is important to recognise that the role of a practice educator encompasses more than that of a mentor in a practice placement for higher education students and raises questions about whether PEMs truly understand their multifaceted role.

Students discussed how they perceived mentors measuring their expectations based on clinical ability. They also predicted that these expectations would become increasingly challenging to meet over their three years of study. In contrast, PEMs stated that they measured their expectations of students based on the students' life experiences and previous work experience. This difference in the measurement of expectations may be related to a lack of confidence in students' clinical abilities and their understanding of the PAD. Potentially, this creates an uneven playing field between students and PEMs, potentially leading students to perform according to incorrect or misperceived expectations.

## Recommendations

To address these issues, the implementation of university-led mentorship modules and financial support for further education emerges as a potential solution. It is important to consider the inclusion of additional mentoring time in scheduling and pay, and collaborative efforts between higher education institutions and the Scottish Ambulance Service to provide necessary support for PEMs. This multifaceted approach ensures that staff, educators, and learners are adequately equipped to engage in effective mentorship within the practice environment.

## Limitations

Whilst this study has heralded important thematic results, further research would benefit from a larger sample size. In addition, independent interviews could potentially yield more candid insights, thereby enriching the depth of the data. It should be acknowledged that opting for codebook TA as a deductive approach may introduce an element of researcher bias, which could potentially influence the remainder of the analysis, for this study that was mitigated by a second researcher reviewing the thematic analysis. Data saturation was reached within the sample; however, the researchers believe data saturation was not reached in answer to the research question. This is due to the small representation of students and mentors in comparison to the Scottish Ambulance Service and higher education student populations within Scotland. Consequently, affecting the transferability of results, it is crucial to consider the inherent constraints associated with this modest sample size and the specific study context.

## Conclusion

The primary aim was to investigate expectations between student paramedics and their mentors. The selected methodology proved effective in identifying key themes through the analysis of data. This is the first primary research looking at the expectations of both practice educators and students of the paramedic profession within Scotland. It has revealed multiple disparities in expectations between these two groups during practice placements, including differences in learning styles, the measurement of expectations, and attitudes toward higher education. Practice placements are crucial for students' professional development as future clinicians, designed to provide opportunities for refining clinical skills, broadening knowledge, and applying theoretical concepts in real-world clinical settings. Any misalignment in expectations within this environment has the potential to greatly affect the teaching and assessment processes within practice education.

This study has raised important questions about how the infrastructure can better prepare for the practice placement of student paramedics. Suggestions include increased investment in support for PEMs, the potential for educational programs that inform about the challenges faced, theories and applications of mentorship, and the creation of stronger links and relationships between higher education institutes and the Scottish Ambulance Service. Further research is required on a larger scale and in alternative settings to determine the extent of the expectation gap, identify how expectations change over time, and generate strategies for overcoming the identified disparities.

## Data Availability

The datasets generated and/or analysed during the current study are not publicly available to uphold participant confidentiality and protect the integrity of ongoing research collaborations but are available from the corresponding author on reasonable request.

## Abbreviations

HCP: Health Care Professional
UK: United Kingdom
TA: Thematic analysis
BSc: Bachelor of Science
HCPC: Health and Care Professions Council
PEM: Practice Educator Mentor
PAD: Practice Assessment Document
UoS: University of Stirling
CoP: College of Paramedics
